# Mouse strain-specific responses along the gut-brain axis upon fecal microbiota transplantation from children with autism

**DOI:** 10.1080/19490976.2024.2447822

**Published:** 2025-01-07

**Authors:** Naika Prince, Lucia N. Peralta Marzal, Léa Roussin, Magali Monnoye, Catherine Philippe, Elise Maximin, Sabbir Ahmed, Karoliina Salenius, Jake Lin, Reija Autio, Youri Adolfs, R. Jeroen Pasterkamp, Johan Garssen, Laurent Naudon, Sylvie Rabot, Aletta D. Kraneveld, Paula Perez-Pardo

**Affiliations:** a Division of Pharmacology, Faculty of Science, Utrecht Institute for Pharmaceutical Sciences, Utrecht University, Utrecht, The Netherlands; b Université Paris-Saclay, INRAE, AgroParisTech, Micalis Institute, Jouy-en-Josas, France; c Faculty of Medicine and Health Technology, Tampere University and Tays Cancer Centre, Tampere, Finland; d Health Sciences, Faculty of Social Sciences, Tampere University, Tampere, Finland; e Department of Translational Neuroscience, UMC Utrecht Brain Center, University Medical Center Utrecht, Utrecht, Netherlands; f Danone Nutricia Research, Utrecht, Netherlands; g Université Paris-Saclay, INRAE, AgroParisTech, CNRS, Micalis Institute, Jouy-en-Josas, France; h Department of Neuroscience, Faculty of Science, Vrije Universiteit Amsterdam, Amsterdam, The Netherlands

**Keywords:** Fecal microbiota transplantation, humanized mouse model, gut-brain axis, autism spectrum disorders

## Abstract

Several factors are linked to the pathophysiology of autism spectrum disorders (ASD); however, the molecular mechanisms of the condition remain unknown. As intestinal problems and gut microbiota dysbiosis are associated with ASD development and severity, recent studies have focused on elucidating the microbiota-gut-brain axis’ involvement. This study aims to explore mechanisms through which gut microbiota might influence ASD. Briefly, we depleted the microbiota of conventional male BALB/cAnNCrl (Balb/c) and C57BL/6J (BL/6) mice prior to human fecal microbiota transplantation (hFMT) with samples from children with ASD or their neurotypical siblings. We found mouse strain-specific responses to ASD hFMT. Notably, Balb/c mice exhibit decreased exploratory and social behavior, and show evidence of intestinal, systemic, and central inflammation accompanied with metabolic shifts. BL/6 mice show less changes after hFMT. Our results reveal that gut microbiota alone induce changes in ASD-like behavior, and highlight the importance of mouse strain selection when investigating multifactorial conditions like ASD.

## 1. Introduction

Autism Spectrum Disorder (ASD) is a set of complex neurodevelopmental disorders characterized by persistent deficits in social communication and interaction, and repetitive patterns of behavior, interests, or activities.^1^ The etiology of ASD is multifaceted with genetic and environmental factors contributing to its development and progression.^2,3^ Besides its manifestations’ heterogeneity, intestinal complaints are frequently described in individuals diagnosed with ASD.^4–6^ Moreover, an increasing number of research conducted in recent years have demonstrated the relevance of the gut-brain axis in regulating brain function and behavior.^7^ Interestingly, the application of microbiota transplantation as therapy has been studied in children diagnosed with ASD showing promising effects improving both intestinal and behavioral complaints related to the disorders.^8,9^ Such evidence supports the theory that changes in gut microbial communities might play an important role in the pathophysiology of ASD.

The presence of microbes in our gastrointestinal tract is essential for our health. For instance, they are a major component shaping early developmental processes of the immune system and brain.^10,11^ Also throughout life, these dynamic and complex populations seem to be relevant regulators of both immunity and behavior.^12^ Thus, the intertwined link between gut microbiota and host’s functions might play a role in the development of ASD when disturbances in the gut microbiota composition and function occur.^13^ Studying how these microbial interactions affect the host is accompanied with several limitations such as the fact that evidence frequently draw associative conclusions rather than causative effects.^14^ In addition, ASD is an early onset condition meaning that most of human studies focus on neurodevelopmental phases including young children, and diagnosis is purely subjective as behavioral tests are primarily used.^15^

One approach to explore the involvement of the gut microbiota in a particular condition is through human fecal microbiota transplantation (hFMT) into animals.^16^ By allowing the recipient’s gut to be colonized by donor’s microbiota, researchers can gain insights into how specific microbial communities influence health and disease phenotypes.^17^ hFMT has been utilized as an experimental setup to assess the effects of microbiota in intestinal and metabolic disorders like inflammatory bowel disease and obesity,^14^ but it has been used also for neurodevelopmental disorders including ASD.^18–21^ Previous studies for autism have mainly employed germ-free mice and have given valuable insights regarding the association between gut dysbiosis and ASD-related outcomes.^19–23^ However, the use of germ-free animals comes with some limitations due to the lack of commensal microbes during developmental stages of life like having an immature immune system, which is one of the major communication pathways in the gut-brain axis.^24^ To overcome this problem, other studies have used microbiota depleted conventional animals.^25^ Besides that, studies performing hFMT into mice for ASD research have shown divergent findings. These discrepancies can be explained by differences in the genetic background of the chosen mouse strain for conducting hFMT, which inherently show biological and behavioral differences. All these emphasize the importance of animal model selection in hFMT studies for ASD investigation.

Our study aims to further explore this variability by employing two distinct mouse strains, BALB/cAnNCrl and C57BL/6J (referred to as Balb/c and BL/6, respectively), chosen specifically for their unique physiological and behavioral profiles. The choice of using microbiota depleted mice is based on using typically developed animals. These mouse strains are known to exhibit marked differences in immune responses,^26–28^ disease susceptibility,^29,30^ and baseline behaviors,^31,32^ making them ideal subjects to investigate the differential impacts of hFMT in the context of ASD. To understand the interplay between genetic predisposition and environmental factors, crucial in ASD research, we conducted a comprehensive analysis investigating gut, brain and systemic parameters. We explored the gut microbiome composition and metabolic function to look at microbial shifts and their host systemic implications. Additionally, we assessed intestinal barrier permeability and mucosal and systemic inflammation, which are essential for better understanding the gut-brain axis’ role in ASD. Most importantly, we performed a series of behavioral tests, including open field and social interaction tests, to evaluate the ASD-like behavioral phenotypes in both mouse strains. Here, our aim is to elucidate the connection between gut microbiota alterations and ASD-like behaviors and physiological changes, and to examine the effects of performing hFMT in mice with distinct genetic backgrounds.

## 2. Materials and methods

### 2.1. Ethical approval and consent

This study was conducted in accordance with institutional guidelines for the care and use of laboratory animals of the University of Utrecht, and approved by the ethical committee (Centrale Commissie Dierproeven; number AVD1080020198547).

The use of fecal material from children was approved by the local ethical commission: Servizio Coordinamento Comitato Etico Campania Sud (approval date 28 February 2019), and written informed consent was obtained from the parents of the donors prior to donating the fecal material to the study.

### 2.2. Human fecal samples

Donors were recruited by the University Hospital of Naples (Italy). Fecal samples from children with ASD suffering from gastrointestinal symptoms and their healthy siblings (Table 1) were collected at home, using the MaaT Pharma stool collection device. Briefly, air was removed from the device immediately after freshly emitted stool collection and stools were transported at 4°C to the lab, where a MaaT Pharma patented diluent containing ascorbic acid and L-cystein as reducing agents was introduced into the device. Five-fold diluted stools were homogenized by malaxing, aliquoted and stored at −80°C.^33^ Aliquots from the same donor group were pooled before hFMT into the mice.

**Table 1.** Characteristics of the fecal donors. *ADOS, autism diagnostic observation schedule; ASD, autism spectrum disorders and gastrointestinal problems; F, female; M, male; NA, not applicable; SD, standard deviation.*

Groups	ASD	Siblings
Age (mean ± SD)	8 ± 1.41	8.25 ± 4.57
Gender (M/F)	4/0	2/2
ADOS score (mean ± SD)	2 ± 1.15	NA
Bristol score (mean ± SD)	1.75 ± 0.96	NA
Food allergies	No	No
Medication		
*Antibiotics*	NA^1^	NA^1^
*Prebiotics*	Neurax Bio (1)	NA
*Other*	Risperidone (1) Valproate (2)	NA

^1^Children were not treated with antibiotics at least 2 months prior fecal sample collection.

### 2.3. Mice

Conventional male BALB/cAnNCrl mice and C57BL/6J mice (3/4 weeks old) were obtained from Charles River Laboratories and the Jackson Laboratory. Mice were group-housed (3 mice per cage) with a 12 h light/dark cycle, with *ad libitum* access to food and water. Mice were fed a standard rodent diet (Ssniff, Germany, V1534–703), and they were sacrificed at the end of the experiment under isoflurane anesthesia. Several tissues were appropriately collected and stored for further analysis.

### 2.4. Experimental set up

**Figure 1.**
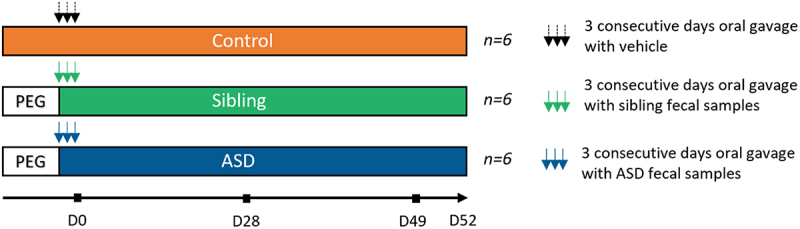
Overview of the experimental design. peg-depleted mice were colonized with fecal samples from sibling or ASD donors on three consecutive days. Transplanted and non-transplanted mice were behaviorally tested at D28 and D49 post-hFMT, and various tissues and samples were collected and stored for further analysis at D52 post-hFMT.

Mice were randomly allocated to three experimental groups for each mouse strain, each experimental group was composed of 6 mice (Figure 1). After arrival, mice were acclimatized to their new environment for one week. Prior to fecal transplantation, the mouse intestinal microbiome was depleted using 1.2 ml of a bowel-cleansing solution containing polyethylene glycol (PEG), as previously described,^34^ when the animals were 4/5 weeks old. After depletion, hFMT was performed, the experimental mice were orally gavaged with freshly pooled stool samples from children with ASD suffering from GI problems or their neurotypical siblings (200 μL inoculum once per day for three consecutive days). Conventional colonized Balb/c (*n* = 6) and BL/6 (*n* = 6) mice received autoclaved tap water via oral gavage.

### 2.5. Behavioral testing

All transplanted mice were tested using the same battery of behavioral tests in the following order: open-field testing, social behavior, and social novelty. To control for time-of-day effects, mice were randomized using Python (v3.6) with the NumPy package. All the sessions were recorded using an overhead camera (The Imaging Source, DMK 22AUC03). All behaviors were analyzed using the EthoVision XT 14 software package (Noldus Information Technology), except for the supported rearing, which was manually scored.

#### 2.5.1. Open field test

The open-field test was performed as previously described in 45 cm^2^ arenas.^35^ The mice were then introduced into the arenas and allowed to explore for 5 min. The total distance traveled, number of entries in the center zone, and number of supported rearing were analyzed.^36,37^

#### 2.5.2. Social interaction test and social novelty test

Consequently, mice were first habituated to two small, perforated Plexiglas cages (10 cm of diameter) for 5 min ^35^. Subsequently, an age- and sex-matched unfamiliar target mouse (target) was introduced into one of the Plexiglas cages. The percentage of time spent interacting with either the empty or the target mouse was calculated. Sociability Index (SI) was calculated as follows:

$$\mathrm{SI}=\frac{\left(\%\mathrm{time}\;\mathrm{with}\;\mathrm{stranger}\;\mathrm{mouse}\right)}{\left(\%\mathrm{time}\;\mathrm{with}\;\mathrm{stranger}\;\mathrm{mouse}+\%\mathrm{time}\;\mathrm{with}\;\mathrm{empty}\;\mathrm{cage}\right)}$$

At the end of the 5 min, each mouse was tested in a 5-min session to measure the social preference for a new target.^38^ The percentage of time spent interacting with either the new target or target mouse was calculated. The ability of the mice to discriminate new social triggers was analyzed by calculating the social recognition index (SRI) as follows:

$$\mathrm{SRI}=\frac{\left(\%\mathrm{time}\;\mathrm{with}\;\mathrm{novel}\;\mathrm{mouse}\right)}{\left(\%\mathrm{time}\;\mathrm{with}\;\mathrm{novel}\;\mathrm{mouse}+\%\mathrm{time}\;\mathrm{with}\;\mathrm{familiar}\;\mathrm{mouse}\right)}$$

### 2.6. Intestinal and brain regions tissue preparation

Intestinal and brain homogenates were prepared as previously described.^39^ The prefrontal cortex (PFC) was isolated, and lysed using a hypotonic solution (10 mm HEPES, pH 7.9, with 1.5 mm MgCl_2_ and 10 mm KCl and Dithiothreitol (0.1 M)) with a protease inhibitor cocktail (1:200; Roche 11,697,498,001). The soluble fraction was collected after centrifugation of the homogenates (20 min at 11,000 × g at 4°C). For the cellular protein fraction, the pellet was resuspended in an extraction buffer composed of RIPA buffer (Thermo Scientific 89,901), dithiothreitol (0.1 M), and a protease inhibitor cocktail (1:200; Roche 11,697,498,001). The cellular protein fraction was collected after centrifugation (5 min at 20,000 × g at 4°C).

Ileal and colonic samples were isolated and stored at −80°C, 15–20 mg of tissues were transferred into homogenization tubes (Precellys lysing kit P000918-LYSKO-A, Bertin Technologies) containing the appropriate volume of RIPA buffer (Thermo Scientific 89,901) with Proteinase Inhibitor Cocktail (1:200; Roche 11,697,498,001). The intestinal tissues were homogenized using a Precellys 24 homogenizer (Bertin Technologies, Montigny-le-Bretonneux, France) three times for 10 s at 6000 rpm speed.

The protein content in each fraction was determined using a Pierce™ BCA Protein Assay Kit (Thermo Scientific 23,225).

### 2.7. Western blot

As previously described,^39^ a total of 20 µg (from cerebral tissue and intestinal tissue) protein was loaded into 4–15% or 4–20% gradient precast polyacrylamide gels (Bio-Rad 5,671,084 or 5,671,094), separated by electrophoresis, and transferred onto PVDF membranes (Bio-Rad 1,704,157). The blots were blocked with 5% milk powder in PBST (0.1% Tween 20 in PBS). Next, the blots were incubated with primary antibodies overnight at 4°C, followed by peroxidase-conjugated goat anti-rabbit or rabbit anti-mouse secondary antibodies (1:2000 or 1:3000; Dako, P0448 and P0260, respectively). Immunoproducts were detected by chemiluminescence with Clarity Western ECL Substrate or Clarity Max Western ECL Substrate (Bio-Rad 1,705,061 or 1,705,062) and were imaged using the ChemiDoc MP Imaging System (Bio-Rad Laboratories, Hercules, CA, USA). The following primary antibodies were used: glial fibrillary acidic protein (GFAP; 1:2000, Dako, Z0334), COX2 (1:500, Cayman 160,107); occludin (1:500; Invitrogen, 40–4700), E-cadherin (1:1000; BD Biosciences 610,182), claudin-3 (1:500; Invitrogen, 34–1700), and GAPDH (1:2000, Cell Signaling, 2118) as loading control.

### 2.8. ELISA

Total protein homogenates from intestinal tissue were used to detect immunoglobulin-A (IgA; Invitrogen, 88-50,450-88). The absorbance was measured using a Promega Glomax Discover plate reader.

### 2.9. Immunostaining

The ileum and colon samples were isolated, fixed in 10% formalin, and embedded in paraffin. Then, samples were sliced into 5 µm sections used for the immunohistochemical analyses.

#### 2.9.1. Chromogenic immunohistochemistry

Staining for serotonergic (5HT^+^) cells started by deparaffinizing the slides. Then, endogenous peroxidase activity was blocked by incubating the slides in 0.3% H_2_O_2_/methanol for 30 min. Next, the slides were rehydrated in serial incubations of ethanol. Afterward, antigen retrieval was performed by incubating the slides in boiling citrate buffer (0.01 M) for 10 min. Blocking was applied by incubating for 30 min the slides with 5% normal goat serum (Dako, X090710–8), 1% bovine serum albumin (BSA) in PBS. Incubation with the primary antibody, rabbit-anti-5HT antibody (1:8000 in 1% BSA/PBS; Sigma, S5545), was performed overnight at 4°C. Then, the slides were incubating for 45 min with the secondary antibody, biotinylated goat-anti-rabbit antibody (1:200 in 1% BSA/PBS; Dako E0432). Subsequently, the slides were incubating for 45 min with the avidin/biotinylated peroxidase complex (VECTASTAIN® ABC-HRP Kit, PK-6100), and stained with DAB (3, 3’-diaminobenzidine; Sigma, D5905) solution for 10 min. Nuclear staining was performed by applying Mayer’s hematoxylin for 30 s. Imaging was performed with the Olympus B×50light microscope using a UPlanFl 20×/0.50 lens and equipped with the Leica DFC 320 digital camera. For each mouse, five pictures were taken and 5HT^+^ cells were counted.

#### 2.9.2. Immunofluorescence immunohistochemistry

Sections were deparaffinized by serial incubations in xylene, decreasing concentrations of ethanol. Antigen retrieval was performed by incubating the slides in boiling citrate buffer (0.01 M) for 10 min. Following an 1 h incubation with serum block (3% BSA/3% normal goat serum/0.1% Tween in PBS), intestinal sections were incubated overnight at 4°C with the appropriate primary antibodies diluted in blocking buffer: rabbit anti-GFAP (1:1000, Dako, Z0334), and mouse anti-E-cadherin (1:200, BD Biosciences 610,182). The intestinal sections were incubated for 1 h with the corresponding secondary antibody diluted in 0.1% BSA/0.1% Tween PBS, goat anti-rabbit AF 594 antibody (1:200, Invitrogen, A11072) and goat anti-mouse AF 488 antibody (1:200, Invitrogen, A11001) Nuclei staining and mounting was performed using ProLong Gold Antifade Mountant with DAPI (Invitrogen, P36931).

Digital images were acquired using either a Leica TCS SP8 confocal microscope with a HCX IRAPO L 25×/0.95 water-immersion lens, or a Keyence BZ9000 microscope with a Plan Apo 20×/0.75 lens. Data were expressed as the number of positive cells per 10 villi. GFAP and E-cadherin expression levels were assessed by measuring the Corrected Total Fluorescence (CTF) for five images per mouse using ImageJ software with the following formula:

$$\mathrm{CTF}=\mathrm{integrated}\;\mathrm{density}-\left(\mathrm{area}\times \mathrm{mean}\;\mathrm{fluorescence}\;\mathrm{of}\;\mathrm{background}\;\mathrm{reading}\right)$$

### 2.10. Flow cytometry

After collection and homogenization of the spleen, single-cell suspensions were used to analyze T cell subsets using flow cytometry as previously described.^39^ Cells (1 × 10^6^ cells per well) were collected in fluorescence-activated cell sorting (FACS) buffer (PBS containing 1% BSA) and plated. The cells were blocked for 20 min with Fc block (anti-mouse CD16/32, Invitrogen, 14-0161-86). Subsequently, cells were stained with the following antibodies in FACS buffer for 30 min at 4°C: anti-CD4-BV510 (BioLegend 100,553), anti-CD69-PE-Cyanine7 (Invitrogen, 25–0691), anti-CD25-PerCP-Cyanine5.5 (Invitrogen, 45–0251), anti-T1/ST2-FITC (mdbioproducts, 101001F), anti-Gata3-PerCP eFluor710 (Invitrogen, 46–9966), anti-Tbet-AF 647 (BioLegend 644,803), anti-RoryT-AF 647 (BD Biosciences 562,682) and anti-FoxP3-FITC (Invitrogen, 11–5773). Cells stained for extracellular markers were fixed using the Foxp3 Staining Buffer Set (Invitrogen, 00-5523-00). The results were collected using a FACS Canto II (BD Biosciences, Franklin Lakes, NJ, US) and analyzed using Flowlogic software (Inivai Technologies, Mentone, Australia).

### 2.11. Metabolomics

Blood samples collected during sectioning were processed and stored as serum at − 80°C until metabolomic analysis. Briefly, metabolites were extracted by diluting 10 μL serum in 1 mL lysis buffer containing methanol/acetonitrile/dH2O (2:2:1). Samples were centrifuged at 16.000 g for 15 min at 4°C to remove cell debris and proteins. LC-MS analysis for metabolite detection was performed on a Dionex UltiMate 3000 LC System (Thermo Scientific) with a thermal autosampler set at 4°C, coupled to a Q-Exactive mass spectrometer (Thermo Scientific). Separation of metabolites was performed using a Sequant ZIC-pHILIC column (2.1 × 150 mm, Merck) with a flow rate of 150 μl/min. A gradient was applied for 20 minutes (elution buffers: eluent A (acetonitrile) and eluent B (20 mm (NH4)2CO3, 0.1% NH4OH in ULC/MS grade water (Biosolve); from 20% eluent A to 60% eluent B). The MS operated in polarity-switching mode with spray voltages of 4.5 kV and −3.5 kV. Data was collected and analyzed using Tracefinder software (Thermo Scientific). Normalization was applied based on total ion count. Single-factor, enrichment and pathway analyses were performed using the platform Metaboanalyst (v6.0).

### 2.12. Microbiome

Cecal content was collected at week 6 post-hFMT to analyze the microbiota composition at the end of the study. Fecal samples were collected at different time points to assess transplantation stability. Fecal and cecal samples were sent to the @BRIDGe platform (GABI, INRAE, AgroParisTech, Paris Saclay-University) for DNA extraction, sequencing library construction, and Illumina sequencing. The following V3-V4 region primers were used for 16S rRNA gene sequencing: forward primer (5’ ACG GRA GGC AGC AG 3’) and reverse primer (5’ TAC CAG GGT ATC TAA TCC T 3’).

The sequences were analyzed using R v.4.2.2, combining dada2 v.1.26,^40^ and FROGS 4.0.0.^41^ The ASVs in the sequence table were then assigned taxonomic names using FROGS affiliation with SILVA v138,^42^ cutoff was set at 95% identity and 100% coverage.^41^ 16S rRNA amplicon sequencing bioinformatic analysis was then performed on those ASVs on R (v4.2.2). Alpha-diversity was explored by the number of observed ASVs, and Chao1, Shannon and inverse Simpson indices. Beta diversity was analyzed using the Bray-Curtis distance for community abundance, and among-group differences were assessed using PERMANOVA, a permutational multivariate analysis of variance by the distance matrices.

### 2.13. Short chain fatty acid (SCFA) analysis

SCFA in fecal contents were measured as previously described in.^43^ Briefly, fecal contents were water-extracted, and proteins were precipitated with phosphotungstic acid. A total of 0.3 µL supernatant was analyzed for SCFA on a gas chromatograph (Agilent 7890B, Agilent Technologies, Les Ulis, France) equipped with a split – splitless injector, a flame-ionization detector, and a capillary column impregnated with SP 1000 (FSCAP Nukol, 15 m × 0.53 mm × 0.5 m) (Supelco, Sigma-Aldrich, Saint-Quentin-Fallavier, France). Obtained peaks were integrated using OpenLAB Chemstation software 2.3.53 (Agilent Technologies). Acetate, propionate, butyrate, and branched and long chain fatty acids (BLCFA; isoSCFAs + valerate + caproate) were expressed as percentages of total SCFAs for statistical analysis.

### 2.14. Quantification and statistical analysis

Differences between groups were statistically analyzed with a t-test, or one-way ANOVA followed by Tukey’s multiple comparisons test. When data were not normally distributed, they were transformed, or a Mann-Whitney test or Kruskal-Wallis test was used. Murine data are presented as mean ± SEM and were considered statistically different when *p* < 0.05. Analyses were performed using GraphPad Prism software (version 9.4.0), and R (version 4.2.2) and *mixOmics* R package.^44^

### 2.15. Integrative analysis

All data from hFMT mice were normalized to no hFMT mice to operate with similar distributions and scale sizes. All the integrative analysis was performed with *mixOmics* R package.^44^ Compositional data, including microbiome at the family level and SCFA, were normalized using Centered Log Ratio (CLR). Metabolites were converted to logarithmic scale. Variables with one missing value were imputed using the Nonlinear Iterative Partial Least Squares (NIPALs) algorithm.^45^ Variables with multiple missing values were filtered from the analysis. One BL/6 mouse from the ASD hFMT group was excluded due to having missing values of the metabolic data. All the biological host responses including gut, spleen and brain measurements by molecular techniques were combined. Subsequent complete data blocks, including tissue, microbiome, SCFA, behavior and metabolites were input to sparse Partial Least Squares – Discriminant Analysis (sPLS-DA). This integrates the data in a supervised manner while simultaneously leveraging the discriminant analysis that takes into account the outcome of ASD hFMT or sibling hFMT, and thus identifies the informative features across the integrated data types that contribute most to the discrimination between these groups.

## 3. Results

### 3.1. hFMT induces mouse strain- and donor-dependent differential abundant bacterial taxa in cecum of transplanted mice

**Figure 2.**
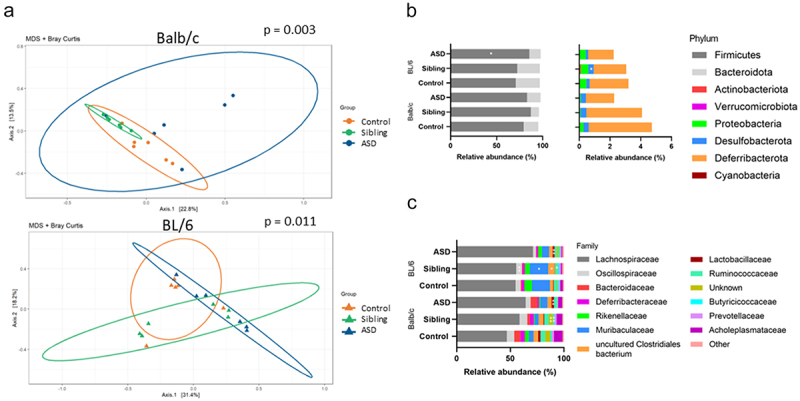
hFMT induces mouse strain- and donor-dependent differential abundant bacterial taxa in cecum of transplanted mice. (a) The first two axes of a multidimensional scaling (MDS) analysis based on Bray-Curtis distances from 16S rRNA gene sequencing of sibling and ASD hFMT mice from both mouse strains including control no hFMT mice. Group differences were tested using pairwise PERMANOVA. (b and c) Cumulative plot bar of mean relative abundances at the phylum and family levels of sibling and ASD transplanted mice from both mouse strains including control no hFMT groups. Mann-Whitney test was applied for comparisons between ASD hFMT and sibling hFMT groups. P-values were adjusted using FDR correction. *P < 0.05, **P < 0.01, (mean ± SEM); *n* = 5–6 mice per group. Cecal samples collected at D52 post hFMT.

To assess the validity of microbiota transplantation, we collected cecal samples from each individual mouse. A shift in α- and β-diversity was observed between human donor and murine recipient microbiota, as demonstrated by others.^46,47^ We observed a loss of bacterial species when transferred to mice, possibly a consequence of sample processing and/or species/host incompatibility. No difference of α-diversity indices between experimental murine groups was observed. On average 52% of bacterial taxa at the genus level in human donor microbiota were present in hFMT mice (Figure S1). Taxa within *Bacteroidota*, *Desulfobacterota*, and *Firmicutes* (more recently referred to as *Bacillota*^48^ thrived whereas others within *Actinobacteriota* failed to grow in the mouse gut, and bacteria belonging to *Verrucomicrobiota* were not detected. β-diversity analysis using Bray-Curtis distance showed that transplanted groups within a mouse strain had different community structures that significantly clustered according to hFMT. Multiple comparisons using PERMANOVA described significant differences between ASD hFMT mice and Sibling hFMT mice in Balb/c strain. However, no significant differences were seen between the hFMT groups of BL/6 mice (Figure 2a). Phylum-level composition in cecal samples of BL/6 mice receiving ASD hFMT was significantly enriched in *Firmicutes* and depleted in *Desulfobacterota* compared to the Sibling hFMT group, but no significant differences were observed in Balb/c transplanted mice (Figure 2b). After applying false discovery rate (FDR) corrections, no significant differences remained (Table S1 and S2). Family-level composition in cecal samples of ASD hFMT mice was significantly enriched in *Lactobacillaceae* and depleted in *Butyricicoccaceae* compared to cecal samples of Sibling hFMT mice in both mouse strains (Figure 2c). In addition, *Oscillospiraceae*, *Muribaculaceae*, and *uncultured Clostridiales* bacteria were significantly reduced in cecal samples of ASD hFMT mice compared to Sibling hFMT mice in BL/6 strain (Figure 2c). After applying FDR correction, significance remained in the observed differences in the Balb/c strain (Table S1 and S2). Transplantation stability was analyzed in fecal samples at different time points along the study timeline. Collectively, cecal samples of mice receiving ASD hFMT or Sibling hFMT harbored distinct microbial profiles.

### 3.2. hFMT induces donor-dependent bacterial metabolic activity shift only in feces of Balb/c mice

**Figure 3.**
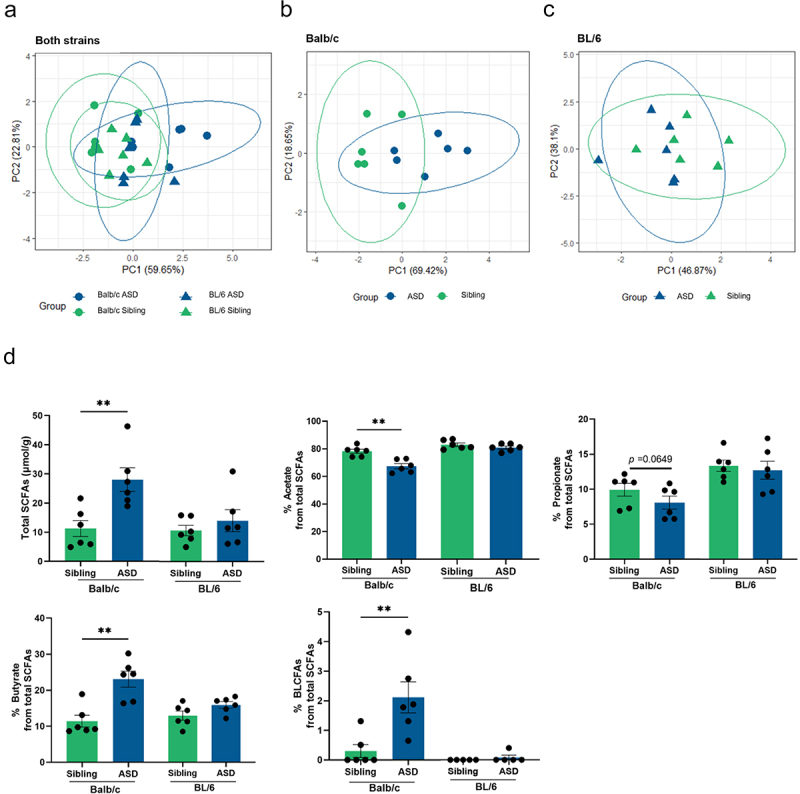
hFMT induces donor-dependent bacterial metabolic activity shift only in feces of Balb/c mice. (a-c) The first two axes of the PCA analysis of bacterial metabolites relative concentrations in sibling and ASD groups in both mouse strains, Balb/c and BL/6 mice. (d) Total short chain fatty acids (SCFAs) including branched and long chain fatty acid (BLCFAs) concentration in murine fecal samples, and percentage of acetate, propionate, butyrate and BLCFAs in Balb/c and BL/6 mice. MANOVA and T-test were used. *P < 0.05, **P < 0.01, (mean ± SEM); *n* = 5–6 mice per group. Fecal samples collected at D52 post hFMT.

To investigate the effect of hFMT on bacterial metabolic activity, fermentation products, including short chain fatty acids (SCFA), i.e., acetate, propionate, butyrate and branched and long chain fatty acids (BLCFA), were measured in fecal samples. There was no significant effect of the mouse strain on fecal SCFA concentrations (Figure 3a), but there was a general effect of hFMT. Balb/c mice transplanted with ASD stools displayed a significantly altered SCFA profile (Figure 3b), while this was not observed in BL/6 mice (Figure 3c). Fecal samples from Balb/c mice transplanted with ASD stools showed a higher proportion of total SCFA, butyrate and BLCFA, and a lower proportion of acetate compared to mice colonized with stools from Sibling donors (Figure 3d). These data indicate that differences in cecal microbial communities between Balb/c mice receiving ASD and Sibling hFMT can lead to different bacterial metabolic activity.

### 3.3. hFMT induces donor-dependent metabolic changes in serum of both Balb/c mice and BL/6 mice

**Figure 4.**
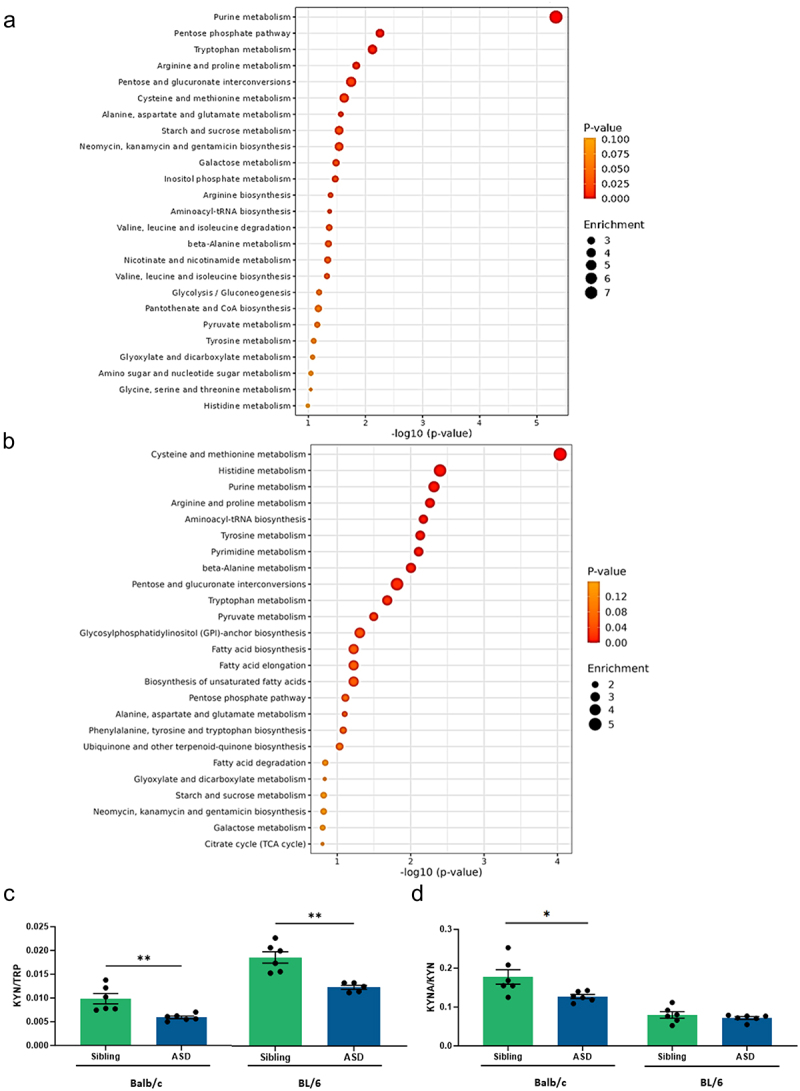
hFMT induces donor-dependent metabolic changes in serum of both Balb/c mice and BL/6 mice. Systemic metabolites of both mouse strains, BL/6 and Balb/c, transplanted with stools from either children with ASD or their siblings was studied by LC-MS. Enrichment analysis was performed and results are represented in a dot-plot showing the 25 more relevant pathways for (a) Balb/c mice, and (b) BL/6 mice. Color intensity reflects statistical significance, while circle diameter shows pathway impact. Additionally, kynurenine pathway was further investigated by calculating (c) KYN/TRP and (d) KYNA/KYN ratios. *P < 0.05, **P < 0.01, (mean ± SEM); *n* = 5–6 mice per group.

Microbial and host metabolism is an important pathway to investigate in ASD as increasing evidence points to their involvement in the disorder.^49^ By using LC-MS in serum samples of Balb/c and BL/6 transplanted mice, we measured 78 and 107 metabolites, respectively. From univariate analysis of the total number of measured metabolites, 12 and 14 compounds were significantly differentiated between ASD and sibling hFMT Balb/c and BL/6 mice, respectively (Table S3 and S4). Also, functional enriched pathway analysis was performed identifying 12 and 11 metabolic pathways significantly altered between ASD and Sibling hFMT Balb/c and BL/6 mice, respectively. To consider that a metabolic pathway was relevant and distinct between the experimental groups, two requirements had to be fulfilled: unadjusted *p* value lower than 0.05, and an enrichment value higher than 2.5 (Table S5 and S6). The most impactfully changed pathways in BL/6 ASD hFMT mice compared to Sibling hFMT mice included: cysteine and methionine metabolism; histidine metabolism; purine metabolism; arginine and proline metabolism; aminoacyl-tRNA biosynthesis; tyrosine metabolism; pyrimidine metabolism; beta-alanine metabolism; pentose and glucuronate interconversions; tryptophan metabolism; pyruvate metabolism; and glycosylphosphatidylinositol-anchor biosynthesis. In Balb/c mice the significantly differentiated pathways were: purine metabolism; pentose phosphate pathway; tryptophan metabolism; arginine and proline metabolism; pentose and glucuronate interconversions; cysteine and methionine metabolism; alanine, aspartate and glutamate metabolism; starch and sucrose metabolism; neomycin, kanamycin and gentamicin biosynthesis; galactose metabolism; and inositol phosphate metabolism (Figure 4a,b). The kynurenic pathway, a major catabolic route from the amino acid tryptophan, is suggested to be relevant in the context of ASD.^50^ Therefore, the ratios kynurenine:tryptophan (KYN/TRP) and kynurenic acid:kynurenine (KYNA/KYN) were calculated to further investigate this pathway. Both KYN/TRP and KYNA/KYN were significantly lower in Balb/c ASD hFMT mice compared to sibling hFMT mice, while in BL/6 hFMT mice, only KYN/TRP was significantly changed, being lower in the ASD hFMT group compared to the sibling hFMT group (Figures 4c,d). Kynurenine concentration in serum of mice that received hFMT from children with ASD was lower than in mice transplanted with stools from the neurotypical siblings. However, tryptophan concentrations were similar in both groups indicating that the decreased KYN/TRP ratio was due to a reduction in KYN metabolization.

### 3.4. hFMT induces mouse strain- and donor-dependent intestinal changes in mice

Increased gut barrier permeability leads to not only intestinal problems, but also other organ-associated detrimental effects often mediated by changes in the immune system.^51^ In ASD, evidence has shown that the gut barrier structure is compromised, allowing the translocation of bacterial parts or products to the blood stream which, in consequence, can travel to the brain and trigger abnormal responses.^52,53^ Here, we investigate the intestinal barrier integrity because of its relevance as a major contributor in the communication between brain and gut.

**Figure 5.**
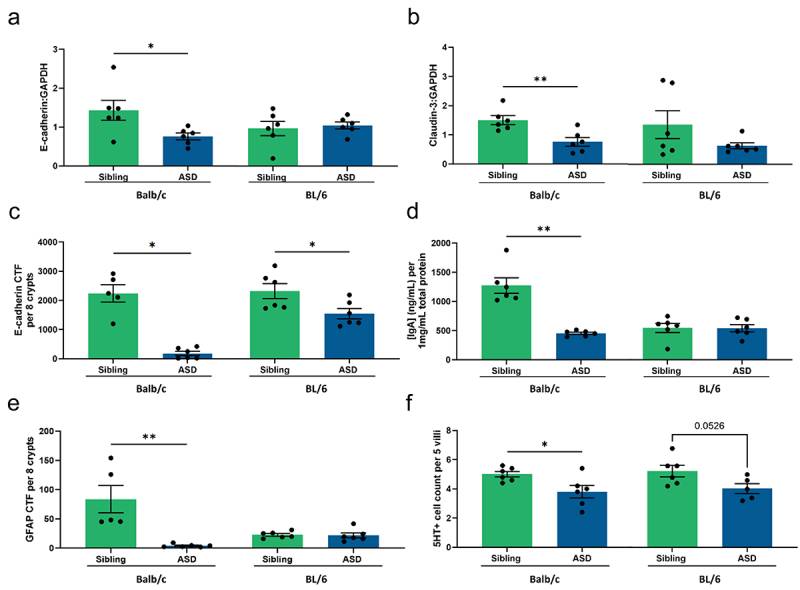
hFMT induces mouse strain- and donor-dependent intestinal changes in mice. Intestinal barrier integrity in the proximal colon of both mouse strains, BL/6 and Balb/c, transplanted with stools from either children with ASD or their siblings, was studied by protein quantification of structural proteins (a) E-cadherin and (b) Claudin-3 relative to GAPDH. In addition, (c) fluorescence immunohistochemistry of paraffin-embedded colonic samples was used to measure E-cadherin expression corrected total fluorescence (CTF) values per 8 crypts in the colon. Lastly, (d) mucosal intestinal immunity was investigated by IgA quantification in the proximal colon of the experimental mice. We assessed ENS function by quantifying (e) GFAP expression, a marker for enteric glial cells, measured as CTF values per 8 crypts, and (f) serotonin-producing cell count per 5 villi in the ileum. T-test or Mann-Whitney test were used for statistical analysis. *P < 0.05, **P < 0.01, (mean ± SEM); *n* = 5–6 mice per group.

In this study, we measure different proteins relevant for forming tight and adherens junctions in ileum and colon of mice transplanted with stools either from children with ASD or their siblings. Both E-cadherin and claudin-3 levels were significantly lower in the colon of ASD transplanted Balb/c mice compared to the sibling group. No differences were found between the hFMT donor groups in the colon of BL/6 mice (Figures 5a,b). E-cadherin was also studied by immunohistochemistry measured as corrected total fluorescence (CTF). Both strains showed lower intensity when the mice were transplanted with stools from children with ASD in comparison to the sibling control groups (Figure 5c). No differences were found in the ileum of both mouse strains after hFMT (data not shown).

In addition, we investigated the intestinal immune environment by measuring IgA levels and enteric glial cells in the intestine of ASD and sibling transplanted mice. We observed a significant reduction in both IgA concentration and glial fibrillary acidic protein (GFAP) intensity, a general marker for glial cells, in the colon of mice transplanted with stools from children diagnosed with ASD in comparison to mice transplanted with stools from their siblings. These differences were only seen in Balb/c mice, while BL/6 mice did not show any significant differences (Figure 5d). No differences were observed in ileum (data not shown). Enteric glial cells found along the gastrointestinal tract in the myenteric and submucosal plexuses are important regulating the enteric nervous system (ENS) and maintaining intestinal homeostasis.^54^ Additionally, enteroendocrine cells in the gut modulate intestinal function by hormone production such as the neurotransmitter 5-HT, which evidence suggests its involvement in ASD.^55^ GFAP, a marker for enteric glial cells, was measured by immunohistochemistry as CTF values. While no differences were seen in the BL/6 mice, Balb/c mice showed lower CTF of GFAP when transplanted with ASD samples compared to Sibling controls (Figure 5e). Additionally, we studied the number of 5-HT-producing cells in the ileum and colon of mice transplanted with stools from either children with ASD or their sibling. A significantly reduced number of 5-HT-producing cells was observed in the ileum of Balb/c mice transplanted with ASD compared to the sibling transplanted group. Similarly, ASD transplanted BL/6 mice did show a decreasing trend in the 5-HT-producing cell count in ileum compared to siblings stool transplanted mice (Figure 5f). No differences were observed in colon of both mouse strains (data not shown). Altogether, these data indicate changes in intestinal barrier integrity, mucosal immunity and ENS function after hFMT of fecal samples from children with ASD compared to their neurotypical siblings in a strain-dependent manner.

### 3.5. ASD hFMT decreases T effector lymphocytes in the spleen of mice

**Figure 6.**
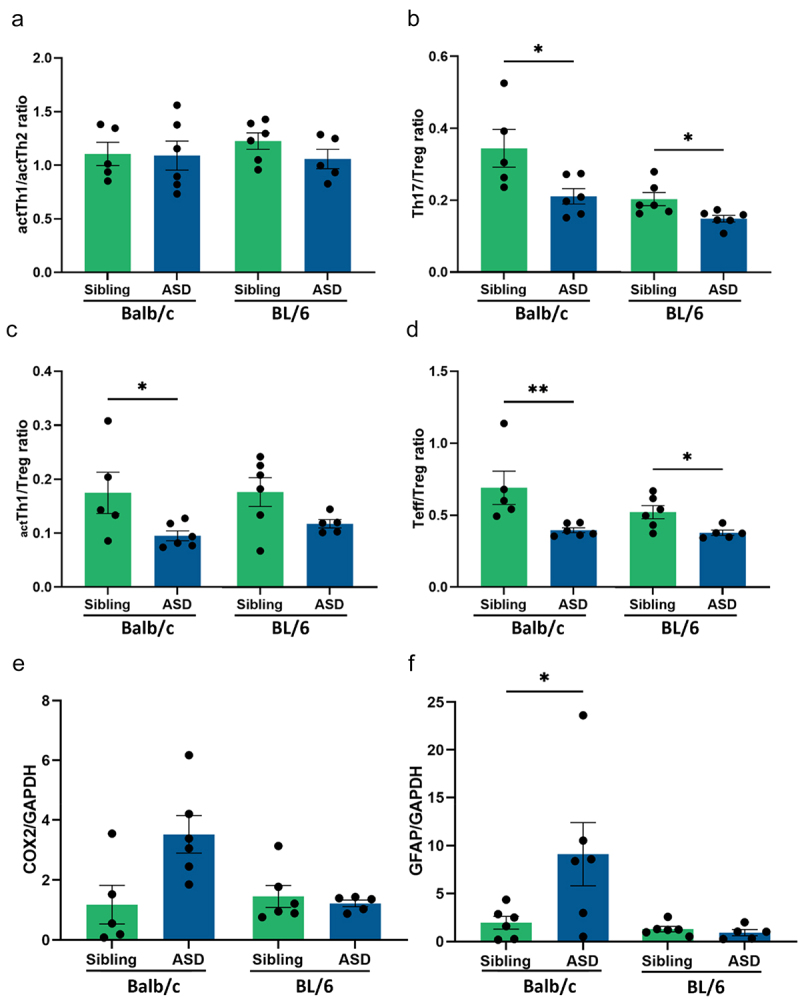
ASD hFMT decreases T effector cells in the spleen of both mouse strains and increases neuroinflammation in the prefrontal cortex only in Balb/c mice. Flow cytometry analysis of T cell populations in the spleen at the end of the study, (a) ratio of actTh1/actth2 cells, (b) ratio of Th17/Treg cells, (c) ratio of actTh1/treg, and (d) ratio of Teff/Treg cells, where Teff includes actTh1, actTh2 and Treg cells. Assessment of neuroinflammation markers in the PFC: (e) COX2 protein expression relative to GAPDH expression, and (f) GFAP protein expression relative to GAPDH expression. T-test or Mann-Whitney test were used for statistical analysis. *P < 0.05, **P < 0.01, (mean ± SEM); *n* = 5–6 mice per group.

The immune system is described as a crucial factor in gut-brain communication and altered in individuals diagnosed with ASD.^56^ Several studies showed dysregulated T helper (Th) cell levels and responses.^57–59^ Therefore, the impact of hFMT on T cell populations was assessed by flow cytometry analysis of splenocytes isolated from mice. Compared to sibling transplanted mice, hFMT from children with ASD did not change the cell balance of activated (act) Th1/Th2 cells in the spleen, regardless of the mouse strain (Figure 6a). However, a significant decrease in Th17/regulatory T cell (Treg) and _act_Th1/Treg cell ratios was observed in ASD hFMT Balb/c mice compared to sibling hFMT group (Figures 6b,c). In addition, ASD hFMT BL/6 mice showed a significant decrease in Th17/Treg cell balance compared to mice receiving hFMT from siblings (Figure 6b); however, no difference was observed in the _act_Th1/Treg cell ratios (Figure 6c). The effector T (Teff)/Treg cell ratio from both mouse strains showed a significant decrease in ASD hFMT mice compared to sibling hFMT mice (Figure 6d).

### 3.6. ASD hFMT increases neuroinflammation in the prefrontal cortex only in Balb/c mice

Individuals diagnosed with ASD show immune activation in different brain areas, including the prefrontal cortex (PFC), a brain region involved in social behavior and cognition.^60,61^ To assess the presence and degree of neuroinflammation, the protein expression of COX2, which is stimulated by proinflammatory cytokines, and GFAP, a glial marker of immune cells, was measured in the PFC. Our results showed a significant increase in COX2 (Figure 6e), and GFAP (Figure 6f) protein expression when Balb/c mice received hFMT from children with ASD compared to the sibling transplanted group, whereas there were no significant differences in both neuroinflammatory markers of hFMT BL/6 mice. Taken together, our data on T cells in spleens and inflammatory markers in PFC indicate an immune dysfunction in response to ASD hFMT, being more pronounced in Balb/c mice.

### 3.7. ASD hFMT decreases explorative activity and social behavior only in Balb/c mice

**Figure 7.**
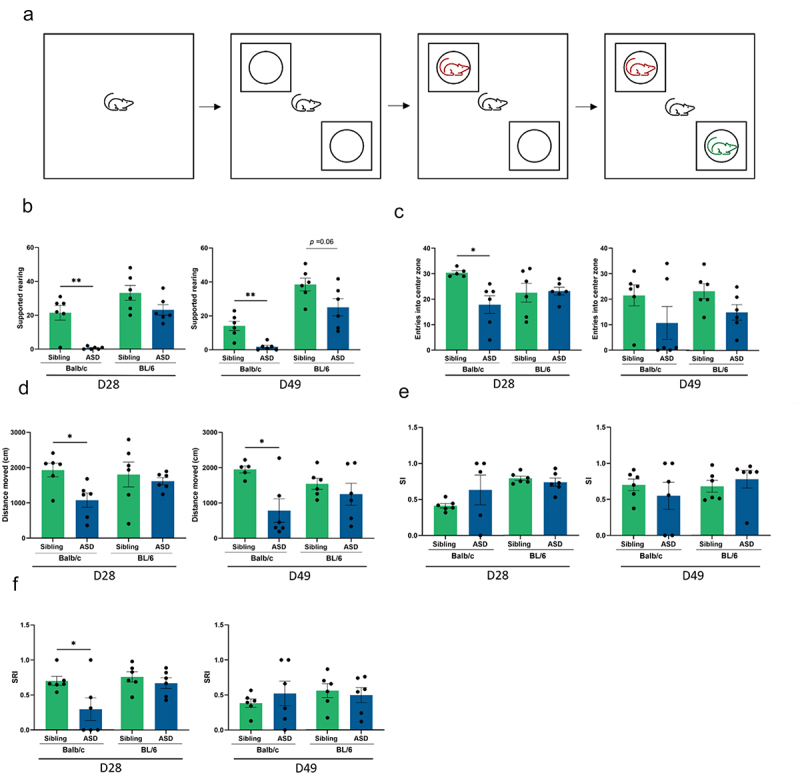
ASD hFMT decreases explorative activity and social behavior in Balb/c transplanted mice. (a) Overview of the behavioral setup to assess exploratory and social behaviors. Experimental mouse (black color), unfamiliar age-matched strain-matched mouse for social interaction (target, red color) and unfamiliar age-matched strain-matched mouse for social novelty (new target; green color). (b) Supported rearing behavior, (c) number of entries in the center zone, and (d) total distance moved were measured. Social behavior assessed using (e) Sociability index (SI) and (f) social recognition index (SRI). T-test or Mann-Whitney test were used for statistical analysis. *P < 0.05, **P < 0.01, (mean ± SEM); *n* = 5–6 mice per group.

Previous human and animal studies have shown that, in addition to social deficits, exploratory behavior is also altered in ASD.^62,63^ Here, we assess exploratory behavior and anxiety-like behavior using the open field test at two time points in the study: at days D28 and D49 post-hFMT. In Balb/c mice only, hFMT with ASD stools decreased vertical exploration (Figure 7b) at both D28 and D49, reduced entry frequency in the central zone (Figure 7c) at D28, and shortened distance walked (Figure 7d) at D28 and D49 compared to hFMT with stools from sibling. Conversely, explorative activity remained unchanged in BL/6 mice after hFMT with stools from children with ASD compared to Sibling. Furthermore, regardless of the mouse strain, no significant differences in sociability were found in ASD hFMT mice compared to siblings hFMT mice at either time point (Figures 7e,f). However, a transient reduction in social recognition index (SRI) was observed on D28 post-hFMT in Balb/c mice transplanted with ASD fecal samples when compared to the sibling transplanted mice (Figure 7f).

### 3.8. Correlative analysis supports the involvement of the microbiota-gut-brain axis in ASD-like phenotype

**Figure 8.**
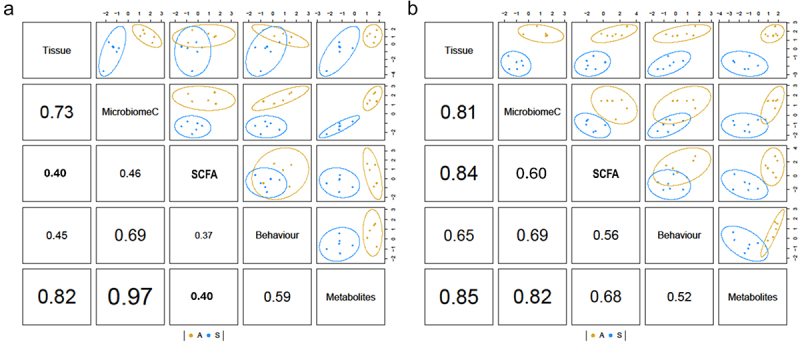
Correlative analysis supports the involvement of the microbiota-gut-brain axis in asd-like phenotype. Representation of correlation values and 2D discrimination plots of integrative analysis after dimensionality reduction technique of the data in (a) BL/6 and (b) Balb/c transplanted mice. Components are formed so that they maximize the correlation between the different datatypes as well as the phenotype. The data block named as *tissue* includes all molecular outcomes measured in gut, spleen and brain representing host’s biological responses.

Univariate statistical analyses fail to depict the complete situation when studying complex conditions like ASD, with still not understood molecular mechanisms, lack of standardization for its diagnoses, and highly variable outcomes, among other limitations. Additionally, microbiome analyses go together with high-dimensional and compositional data, also inconvenient when using conventional data analysis.^64,65^ To overcome this and being able to have a clearer overview describing the interplay between different components relevant for ASD, we performed data supervised integration using generalized Projection to Latent Structures dimensionality reduction- so that the correlation between the datasets and the hFMT outcome is maximized (see section 2.15.). All the biological host’s responses including gut, spleen and brain measurements by molecular techniques were combined in the component named as to *tissue*. Interestingly, we reported a correlation of 0.69 for both strains transplanted with fecal samples either from children with ASD or their siblings between gut microbiome and behavioral outcomes. Also, it is important to highlight that the highest correlation of gut microbiome was the metabolic profile of Balb/c and BL/6 transplanted mice with values of 0.82 and 0.97, respectively (Figure 8a,b). Together, we can conclude that there is a great impact of hFMT in behavior, and that it can be mediated via changes in metabolism.

## 4. Discussion

In the last years, the use of animal experimentation plus hFMT has become a powerful tool to investigate the effects of complex communities of microorganisms colonizing the gut in health and disease. Most studies employing mouse strains to investigate the effects of hFMT from children with ASD used germ-free mice. However, some of the limitations using germ-free mice involve the abnormal development of their immune system as well as brain.^66^ Here, we performed for the first time hFMT into gut microbiota depleted conventional mice for ASD research. The choice of the widely used mouse strains, BL/6 and Balb/c, was to investigate whether the genetic background might play an important role in the effects triggered by hFMT intervention. It is important to note that hFMT into mice has different approaches. While there is no data regarding gut microbiome post-PEG intervention in our study, there is evidence indicating that bowel cleansing is a successful method to deplete murine microbiota allowing further engraftment of human microbes.^67,68^ Microbiota-depletion by bowel cleansing was performed following Le Roy et al. (2019) were they show that PEG administration for bowel cleansing is more efficient in juvenile mice using 1.2 mL of a PEG solution (PEG 77.5 mg/mL).^34^ However, higher dosages of PEG have been described to be necessary for depleting microbes in the gut of older mice.^14,68^ Hence, mouse age and PEG concentration are important factors to take into account when performing bowel cleansing. While our study performed hFMT with pooled fecal samples, other studies can be designed transplanting one donor’s samples into one or several recipient mice.^14^ Recently, two enterotypes have been described to be linked to ASD, with one of them being associated with more pronounced social deficits.^69^ Considering that different subtypes based on microbiome might be related to different ASD outcomes is something that should be further studied. Although one-to-one hFMT might offer a better individual representation of the phenotype, combining samples from autistic children might account for the high heterogeneity of ASD.

One of the most striking outcomes from our results was that hFMT from children with ASD was sufficient to trigger behavioral differences in mice compared to the mice receiving sibling hFMT. This aligns with previous literature suggesting a critical role of gut microbiota in modulating behavior.^24,70^ The results show a mouse strain-dependent effect on behavior, where hFMT from children with ASD significantly impaired explorative, anxious and social recognition behaviors in Balb/c mice, associated with altered bacterial composition and activity, decreased gut barrier permeability, changed metabolic pathways and dysregulated immune homeostasis, whereas no or limited effects were observed in BL/6 mice after ASD hFMT. This emphasizes the intricate interplay between genetic predispositions, gut microbiota, and behavioral outcomes.

Microbiota composition and metabolite availability in the gut are the main contributors to the production of bacteria-specific metabolites like SCFAs. These compounds have shown to exert neuroactive properties and are often associated with beneficial effects for the host. Gut dysbiosis can trigger metabolic changes that, in turn, lead to neuronal deficits indicating their relevance in neurological disorders as ASD.^71,72^ In Balb/c, the *Lactobacillaceae* family showed an increase while the *Butyricicoccaceae* family exhibit a decrease in the cecum of ASD hFMT mice. Bacterial species belonging to both families are known SCFA-producers, thus the differentiated bacterial metabolism between ASD and Sibling hFMT in feces of the Balb/c strain can be explained by a bacterial taxa shift. Nevertheless, studies of ASD have reported inconsistent results regarding fecal SCFA levels, with some showing no differences,^73^ others indicating higher proportions,^74^ and still others finding lower levels^75,76^ when compared to neurotypical individuals. Also, comparing fecal SCFA concentrations to bacterial composition in the cecum may not be representative as the vast metabolization of SCFA takes part in the cecum and proximal colon, and their absorption at the distal colon.^77^ Although bacterial differences in the gut are accompanied with differential bacterial metabolism, more extensive research is needed to directly link these metabolic changes to the behavioral phenotypes observed in ASD. Another limitation of this study is the absence the absence of Bristol Stool Score data within the sibling group that might constitute a confounding factor, as stool consistency is known to influence microbiota composition. Including this measure would have allowed a more precise comparison of gastrointestinal characteristics between groups, reducing potential variability attributable to differences in stool consistency. Additionally, the lack of reproducibility when comparing outcomes across different clinical and preclinical studies (referring to microbiome studies in ASD) can be partially explained by the need of standardization of the methods (e.g., fecal samples collection, sequencing technique and pipeline used for microbiota analysis), controls’ selection (neurotypical unrelated children or neurotypical siblings, age-matched, etc.), and a more thorough track of information, for both ASD and non-ASD individuals, like lifestyle, dietary preferences, nutritional supplementation, and gastrointestinal complaints, among other factors that are relevant for the disorder and gut-brain axis’ responses.

Another pivotal aspect of our study was the observation of decreased gut barrier permeability and dysregulated mucosal immune homeostasis mostly in Balb/c mice following ASD hFMT compared to the sibling hFMT mice. This phenomenon indicates that the impact of hFMT extends beyond alterations in the microbial composition and it significantly influences multiple host’s intestinal physiological responses. Intestinal barrier, mucosal immunity and the ENS are key factors to consider when studying the influence of gut microbiota in health and disease.^78,79^ These are greatly influenced by gut microbiota being frequently compromised when there is gut dysbiosis.^80–82^ In our study, we show that hFMT with ASD stools disrupts the epithelial barrier shown by a reduction in E-cadherin and claudin-3 protein levels; these differences were mostly observed in Balb/c transplanted mice. Also, mucosal barrier in the colon, measured as IgA concentration, is compromised in the ASD hFMT Balb/c mice compared to the sibling hFMT mice. It is known that intestinal immunity is maintained by IgA among other factors, and that specific bacteria in the gut are positively or negatively linked to its levels.^83^ The ENS is affected by the ASD hFMT as we observed a strong decrease in GFAP intensity in the colon of Balb/c transplanted mice indicating a malfunction of the enteric glial cell system. Multiple biological processes like intestinal function, endocrine system and neuronal modulation are controlled by these heterogeneous set of enteric cells.^84^ Altogether, we demonstrate that hFMT compromises the intestinal barrier and immune homeostasis by introducing certain microbial communities that are associated with ASD specially in Balb/c recipient mice.

The disparity in behavioral responses after transferring a group of microbes can be explained by the broader framework of mouse strain-specific predispositions.^85,86^ One of the troubles when using animal models for behavioral studies is the variation that the different mouse strains display by themselves.^87^ Besides behavioral differences between mouse strains, immune dissimilarities are frequent seen at strain, even sub-strain, level.^88,89^ Balb/c mice are known for their propensity toward a Th2 cell-mediated immune response, while BL/6 mice are characterized by dominant Th1 cell-mediated responses.^90^ Then, the different T effector cell profile might be an explanation of being more susceptible or resilient to hFMT. Differential susceptibilities to Treg-mediated suppression among these strains,^91^ offer a potential explanation for the distinct behavioral outcomes observed. This is further substantiated by literature indicating the role of IL-17 a cytokine in promoting sociability in mouse models of neurodevelopmental disorders.^92^ The decreased ratio of Th17/Treg cells in the spleens of ASD hFMT mice, therefore, may have contributed to the observed behavioral deficits, reinforcing the significance of the gut-immune-brain axis in ASD. Also, systemic immunity is tightly link to brain immunity. Our findings show neuroinflammation in the PFC of Balb/c transplanted mice receiving stools from children with ASD when compared to their neurotypical siblings. This brain region is relevant for social and executive functions, in addition to regulating exploratory behavior.^93–95^ Knowing that immune events modulate brain functions like sociability,^96^ we can point to a pivotal influence of the immune system of the mouse strains’ behavioral outcomes.

Gut microbiota composition and function are actively involved in multiple host’s physiological responses including systemic metabolism, which, in addition, regulates processes all over the body, including brain function.^97^ In ASD, there is a growing body of evidence for the implication of metabolic changes, often associated with gut dysbiosis.^98,99^ Our study has identified relevant metabolic pathways that are differentiated in the serum of ASD and Sibling hFMT mice for both strains. Interestingly, one of the most important pathways for both strains is purine metabolism showing an increased proportion of metabolites involved in this metabolic route in ASD hFMT mice. The final product of purine catabolism is uric acid, which, in physiological concentrations, acts as an antioxidant compound. Several studies observed lower levels of uric acid in individuals with ASD, which might indicate an oxidative imbalance. However, hyperuricemia has been also described in ASD and psychiatric disorders.^100,101^ Pyrimidine catabolism produces as end-product beta-alanine, a precursor of carnosine together with histidine. We observed a reduction in both metabolites in the ASD hFMT groups of Balb/c and BL/6 mice, respectively. Carnosine promotes correct brain functioning acting as an antioxidant compound, and a reduction of this metabolite has been previously described in the urine of children diagnosed with ASD and presenting gastrointestinal complaints compared to control children.^102^ Moreover, carnosine supplementation has been shown to improve ASD-related behavioral problems such as hyperactivity and sociability.^103^

In addition to purine metabolism, our data show that arginine, proline, cysteine, and methionine metabolism is strongly influenced by hFMT. Interestingly, all the aforementioned pathways have a commonality, which is the methylation cycle. This process involves the folate and methionine cycle and is essential for many biological pathways in the host including redox homeostasis, immune response, and neurodevelopment.^104–106^ Furthermore, gut microbiota can directly or indirectly modulate the host’s methylation.^107,108^ Methylation status can be studied by investigating S-adenosylmethionine (SAM) and S-adenosylhomocysteine (SAH). Most of previous studies point to a compromised methylation function given by reduced levels of methionine, SAM and SAM/SAH ratio in ASD.^105,109^ On the contrary, we did not find any differences in methionine concentration or SAM/SAH ratio, and we did report a significant increase of both SAM and SAH compounds in ASD hFMT compared to the sibling hFMT BL/6 mice. In ASD hFMT Balb/c mice, we detected lower concentrations of arginine compared to sibling hFMT mice. Arginine is highly relevant for detoxifying ammonia, an active compound associated with detrimental effects in the immune system and CNS.^110^ Also, arginine degradation produces nitric oxide, among other compounds, which acts as a signaling molecule regulating multiple biological processes. However, abnormally higher levels of nitric oxide have been associated with ASD.^111^

Other implications of the folate cycle in ASD can be mediated by tetrahydrobiopterin, a compound involved in neurotransmitter metabolism as dopamine and serotonin synthesis.^112^ Both neurotransmitters are essential during the neurodevelopmental phase, and their abnormal function has often been linked to ASD.^113,114^ Serotonin production uses tryptophan as precursor molecule. Tryptophan and its derivatives are highly relevant for neurodevelopmental processes, and their imbalances are associated to brain dysfunction like inflammation, and behavioral impairments such as cognitive deficits and anxiety-like behaviors.^115^ A recent study performing hFMT from children with ASD into germ-free mice described alterations in the tryptophan and serotonergic systems possibly associated to specific changes in the gut bacteria.^21^ However, the direct and molecular mechanisms remain unclear. Interestingly, tryptophan can undergo other additional metabolic pathways- the kynurenine pathway and gut-bacterial metabolism into indolic compounds.^116,117^ Many studies have reported abnormalities in the kynurenine pathway of individuals affected by ASD linked to immune disbalance, thus providing evidence of the relevance of kynurenine and derivative molecules in the condition.^118^ Both mouse strains show a lower KYN/TRP ratio in ASD hFMT mice compared to sibling hFMT mice. These changes are explained by a lower amount of KYN in the ASD hFMT mice compared to the sibling hFMT groups. Similar to previous studies showing a decreased concentration of KYNA, a compound known to exert neuroprotective effects, in the serum of children with ASD,^119^ KYNA/KYN ratio and KYNA were significantly lower in ASD hFMT Balb/c mice compared to the sibling hFMT group. However, the opposite outcome of higher concentrations of KYN and KYNA has been also linked to a mouse model showing ASD-like behavior.^120^ Although we do not have information regarding the systemic levels of serotonin, we observed a decreased production in the ileum of both ASD groups when compared to sibling mice. These results support the hypothesis of an abnormal tryptophan metabolism in ASD.^121^ Nonetheless, additional investigation on how tryptophan is metabolized by the host and gut microbiota, and what the biological consequences are will contribute to a better understanding of the disorder.

Although correlation does not imply causation, associative results can help to target specific processes relevant for a given phenotype. Thus, trying to understand gut microbes’ contribution to ASD, we looked at correlations between the different components analyzed in this study. Surprisingly, high scores were observed between behavioral and microbiome data supporting the theory that emphasizes how impactful microbial communities in the gut are for brain function. Moreover, changes observed after the transplantation of ASD-associated microbes into mice are possibly mediated by metabolic routes. A study limitation is the small sample size of the experimental groups. Even with a limited number of animals, we could detect robust and significant differences in the measured parameters including behavior, systemic metabolites, and gut bacterial communities. However, more powered study designs and further research are still needed to fully unveil the molecular mechanisms behind the pathophysiology of ASD.

This study provides valuable insights into the gut-brain axis’ role in ASD, emphasizing the complex interplay between microbial communities, gut function, host metabolic profile, immune responses, and behavioral outcomes. Our findings highlight the importance of considering genetic variability in future research and the potential for microbiota-based therapeutic strategies in ASD. Future studies should aim to unravel the mechanistic underpinnings of these interactions, focusing on the role of specific microbial taxa, metabolites, and immune pathways in modulating neurobehavioral outcomes in ASD. Additionally, longitudinal studies assessing the direct effects of microbiota alterations on behavior and neurological health are crucial to fully understand the implications of our findings.

## Supplementary Material

Supplemental Material

## Data Availability

Datasets analyzed in this study can be found in Mendeley Data, doi: 10.17632/g9n8s55826.1. Any additional information required to reanalyze the data reported in this paper is available from the Corresponding author, Paula Perez-Pardo (p.perezpardo@uu.nl), upon request.
