# Supplementary material for: Mouse strain-specific responses along the gut-brain axis upon fecal microbiota transplantation from children with autism
Source: Gut Microbes. 2025 Jan 7;17(1):2447822. doi: 10.1080/19490976.2024.2447822 (PMC11730631; doi:10.1080/19490976.2024.2447822)
Supplement: Supplemental Material [file KGMI_A_2447822_SM1743.zip › Supplemental information2ndRevision.docx]

Supplemental information


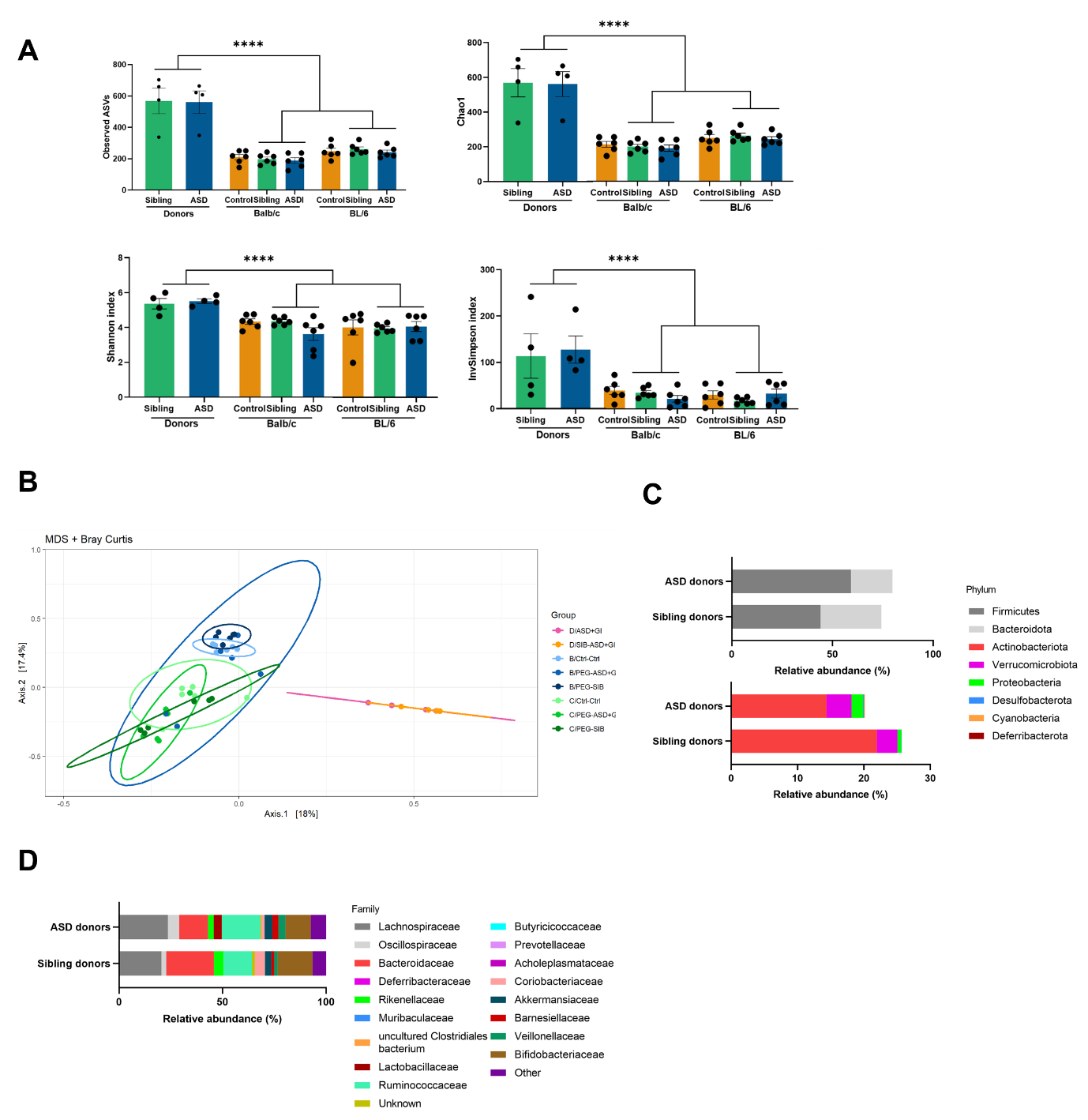


**Figure S1.** Related to Figure 2: hFMT induces mouse strain- and donor-dependent differential abundant taxa in caecum in transplanted mice. (**A**) Bar-plots showing differences of α-diversity between donors (*n* = 4) and recipient mice (*n* = 6) as number of observed ASVs, Chao1, Shannon and inverse Simpson indices. (**B**) Two-dimension plot of a multidimensional scaling (MDS) analysis based on Bray-Curtis distances from 16S rRNA gene sequencing of Sibling and ASD hFMT mice for both mouse strains including control group (no hFMT) and donors. Group differences were tested using pairwise PERMANOVA. (**C** and **D**) Cumulative plot bar of mean relative abundances at phylum and family levels of Sibling and ASD donors.

**
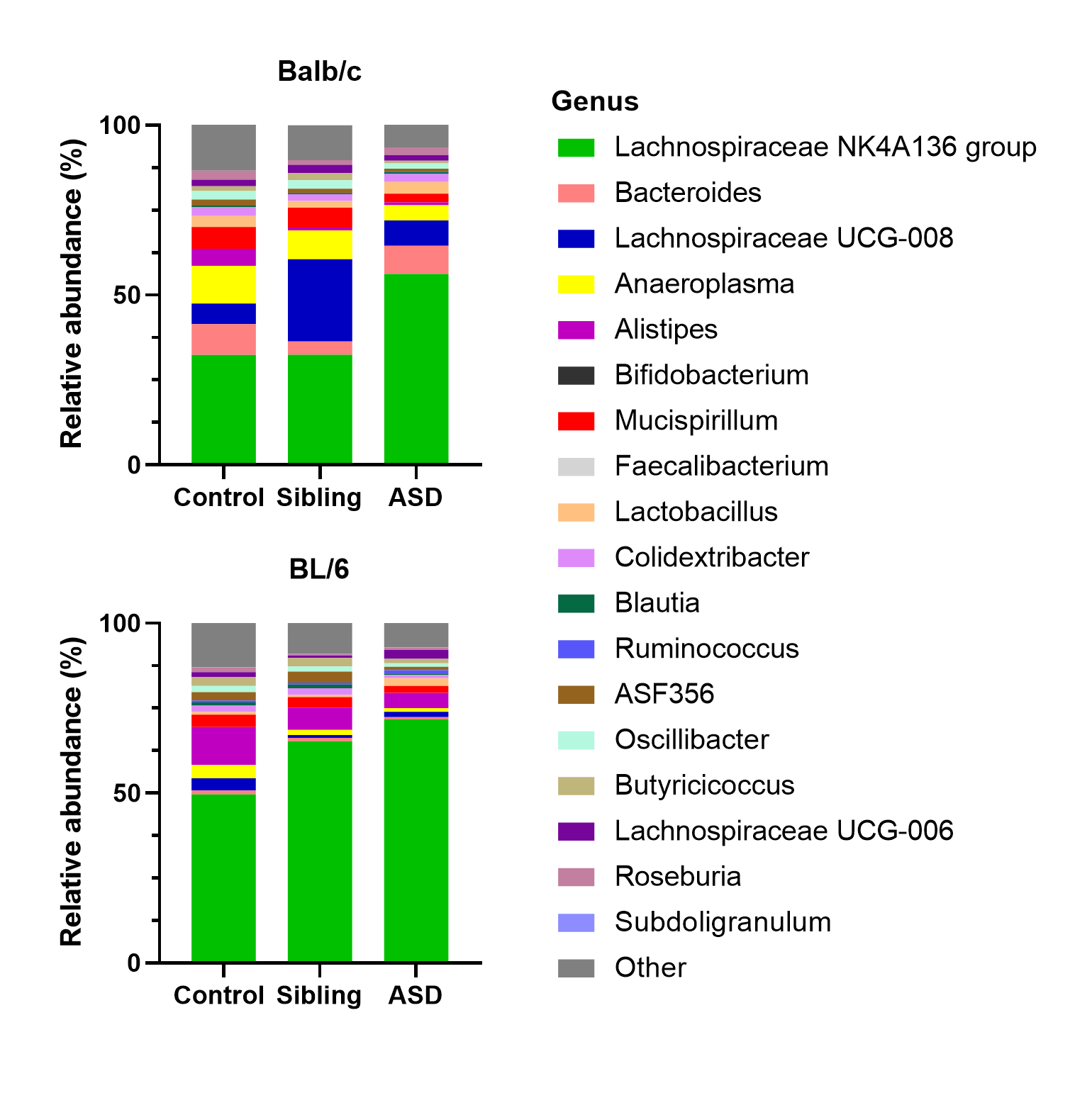
**

**Figure S2.** Related to Figure 2: hFMT induces mouse strain- and donor-dependent differential abundant taxa in caecum in transplanted mice. Cumulative plot bar of mean relative abundances at genus level of control, Sibling and ASD mice (*n* = 6). In Blab/c mice, *Bacteroides* (*p* < 0.01)*, Lachnospiraceae UCG-008* (*p* < 0.0001)*, Anaeroplasma* (*p* < 0.01)*, Mucispirillum* (*p* < 0.05), *Butyricicoccus* (*p* < 0.05) were significantly reduced in ASD mice compared to Sibling, while and *Lachnospiraceae NK4A136 group* (*p* < 0.0001), *Lactobacillus* (*p* < 0.05) were significantly increased. In BL/6 mice, *Lactobacillus* (*p* < 0.05), *Lachnospiraceae NK4A136 group* (*p* < 0.0001) were also significantly increased in ASD mice compared to Sibling, while *Butyricicoccus* (*p* < 0.05), was significantly depleted . Statistical significance was determined using Tukey multiple comparison test.

**Table S1.** Mann-Whitney test analyzing differences on cecal microbiome differences at phylum and family levels between ASD hFMT and Sibling hFMT groups in BL6 mice with FDR correction. Related to Figure 2.

|  | **p value** | **FDR** |
| --- | --- | --- |
| **Phylum** |  |  |
| *Firmicutes* | 0.041126 | 0.172727 |
| *Bacteroidota* | 0.064935 | 0.181818 |
| *Actinobacteriota* | 0.372294 | 0.781818 |
| *Verrucomicrobiota* | >0,999999 | >0,999999 |
| *Proteobacteria* | 0.937229 | >0,999999 |
| *Desulfobacterota* | 0.015152 | 0.127273 |
| *Deferribacterota* | 0.588745 | 0.989091 |
| *Cyanobacteria* | >0,999999 | >0,999999 |
| **Family** |  |  |
| *Lachnospiraceae* | 0.132035 | 0.323485 |
| *Oscillospiraceae* | 0.004329 | 0.063636 |
| *Bacteroidaceae* | 0.937229 | 0.984091 |
| *Deferribacteraceae* | 0.588745 | 0.809917 |
| *Rikenellaceae* | 0.484848 | 0.791919 |
| *Muribaculaceae* | 0.041126 | 0.120909 |
| *uncultured Clostridiales bacterium* | 0.025974 | 0.120909 |
| *Lactobacillaceae* | 0.008658 | 0.063636 |
| *Ruminococcaceae* | 0.818182 | 0.925175 |
| *Unknown* | 0.24026 | 0.504545 |
| *Butyricicoccaceae* | 0.041126 | 0.120909 |
| *Prevotellaceae* | 0.484848 | 0.791919 |
| *Acholeplasmataceae* | 0.606061 | 0.809917 |
| *Other* | 0.818182 | 0.925175 |

**Table S2.** Mann-Whitney test analyzing differences on cecal microbiome differences at phylum and family levels between ASD hFMT and Sibling hFMT groups in Balb/c mice with FDR correction. Related to Figure 2.

|  | **p value** | **FDR** |
| --- | --- | --- |
| **Phyla** |  |  |
| *Firmicutes* | 0.937229 | >0,999999 |
| *Bacteroidota* | 0.818182 | >0,999999 |
| *Actinobacteriota* | 0.545455 | >0,999999 |
| *Verrucomicrobiota* | >0,999999 | >0,999999 |
| *Proteobacteria* | 0.738095 | >0,999999 |
| *Desulfobacterota* | 0.699134 | >0,999999 |
| *Deferribacterota* | 0.093074 | 0.781818 |
| *Cyanobacteria* | >0,999999 | >0,999999 |
| **Family** |  |  |
| *Lachnospiraceae* | 0.24026 | 0.346875 |
| *Oscillospiraceae* | 0.179654 | 0.345833 |
| *Bacteroidaceae* | 0.937229 | 0.825 |
| *Deferribacteraceae* | 0.093074 | 0.215 |
| *Rikenellaceae* | 0.484848 | 0.622222 |
| *Muribaculaceae* | 0.588745 | 0.68 |
| *uncultured Clostridiales  bacterium* | >0,999999 | 0.825 |
| *Lactobacillaceae* | 0.008658 | 0.033333 |
| *Ruminococcaceae* | 0.093074 | 0.215 |
| *Unknown* | 0.004329 | 0.025 |
| *Butyricicoccaceae* | 0.004329 | 0.025 |
| *Prevotellaceae* | 0.699134 | 0.734091 |
| *Acholeplasmataceae* | 0.24026 | 0.346875 |
| *Other* | 0.937229 | 0.825 |

**Table S3.** Univariate analysis. Significantly differentiated metabolites in serum of no hFMT, ASD hFMT or Sibling hFMT BL/6 mice (*n* = 5-6). Related to Figure 4.

| **Metabolite** | **p value** | **FDR** |
| --- | --- | --- |
| Pyruvate | 0.004329 | 0.1443 |
| S-Adenosyl-Homocysteine | 0.004329 | 0.1443 |
| N-Carbamoyl-aspartate | 0.004329 | 0.1443 |
| Guanosine | 0.007969 | 0.1443 |
| IMP | 0.015024 | 0.1443 |
| Histidine | 0.016887 | 0.1443 |
| Butyrylcarnitine | 0.017316 | 0.1443 |
| Glucosamine | 0.017316 | 0.1443 |
| S-Adenosyl-Methionine | 0.017316 | 0.1443 |
| D-Ribose 5-phosphate | 0.017316 | 0.1443 |
| D-Ribulose 5-phosphate | 0.017316 | 0.1443 |
| Inosine | 0.017316 | 0.1443 |
| Glucose | 0.030303 | 0.21645 |
| Adenosine | 0.030303 | 0.21645 |

**Table S4.** Univariate analysis. Significantly differentiated metabolites in serum of no hFMT, ASD hFMT or Sibling hFMT Balb/c mice (*n* = 5-6). Related to Figure 4.

| **Metabolite** | **p value** | **FDR** |
| --- | --- | --- |
| Kynurenine | 0.002165 | 0.077922 |
| Hypoxanthine | 0.003665 | 0.077922 |
| Inosine | 0.003665 | 0.077922 |
| Oleoylcarnitine | 0.004329 | 0.077922 |
| ARGININE | 0.008658 | 0.089054 |
| LEUCINE | 0.008658 | 0.089054 |
| GLUCOSE 6-PHOSPHATE | 0.008658 | 0.089054 |
| HISTIDINE | 0.015152 | 0.12121 |
| Proline | 0.015152 | 0.12121 |
| Pyruvate | 0.025974 | 0.18616 |
| D-Ribulose 5-phosphate | 0.028441 | 0.18616 |
| L-Hexanoylcarnitine | 0.041126 | 0.24675 |

**Table S5.** Pathway analysis. Significantly differentiated metabolic pathways in serum of ASD hFMT or Sibling hFMT BL/6 mice (*n* = 5-6). Related to Figure 4.

| **Metabolic pathway** | **p value** | **FDR** | **Enrichment** |
| --- | --- | --- | --- |
| Cysteine and methionine metabolism | 9.20E-05 | 0.004875 | 5.444 |
| Histidine metabolism | 0.0039783 | 0.058897 | 4.905 |
| Purine metabolism | 0.0048113 | 0.058897 | 4.009 |
| Arginine and proline metabolism | 0.0054592 | 0.058897 | 3.289 |
| Aminoacyl-tRNA biosynthesis | 0.0067115 | 0.058897 | 2.953 |
| Tyrosine metabolism | 0.0074073 | 0.058897 | 3.224 |
| Pyrimidine metabolism | 0.0077789 | 0.058897 | 3.103 |
| beta-Alanine metabolism | 0.0099088 | 0.065646 | 3.441 |
| Pentose and glucuronate interconversions | 0.01534 | 0.090334 | 4.974 |
| Tryptophan metabolism | 0.020848 | 0.11049 | 3.4 |
| Pyruvate metabolism | 0.031851 | 0.15346 | 2.818 |
| Glycosylphosphatidylinositol (GPI)-anchor biosynthesis | 0.049303 | 0.21129 | 3.642 |

**Table S6.** Pathway analysis. Significantly differentiated metabolic pathways in serum of ASD hFMT or Sibling hFMT Balb/c mice (*n* = 5-6). Related to Figure 4.

| **Metabolic pathway** | **p value** | **FDR** | **Enrichment** |
| --- | --- | --- | --- |
| Purine metabolism | 4.63E-06 | 0.000227 | 7.969 |
| Pentose phosphate pathway | 0.00557 | 0.12305 | 3.783 |
| Tryptophan metabolism | 0.007534 | 0.12305 | 4.334 |
| Arginine and proline metabolism | 0.014494 | 0.13612 | 3.105 |
| Pentose and glucuronate interconversions | 0.017791 | 0.13612 | 4.896 |
| Cysteine and methionine metabolism | 0.023578 | 0.13612 | 4.242 |
| Alanine, aspartate and glutamate metabolism | 0.027013 | 0.13612 | 2.308 |
| Starch and sucrose metabolism | 0.02895 | 0.13612 | 3.757 |
| Neomycin, kanamycin and gentamicin biosynthesis | 0.02895 | 0.13612 | 3.757 |
| Galactose metabolism | 0.03274 | 0.13612 | 2.916 |
| Inositol phosphate metabolism | 0.033785 | 0.13612 | 2.741 |
